# Immediate analgesic effect of cervical localized rotation manipulation combined with cervical Huatuo–Jiaji electroacupuncture on cervicogenic headache

**DOI:** 10.1097/MD.0000000000046011

**Published:** 2025-12-12

**Authors:** Chao Zhong, Fang Zhu, Qian Liang

**Affiliations:** a Department of Pain Management, Affiliated Hospital of Chengdu University of Traditional Chinese Medicine (Sichuan Provincial Hospital of Traditional Chinese Medicine), Chengdu, Sichuan Province, China; b Department of Pharmacy, Affiliated Hospital of Chengdu University of Traditional Chinese Medicine (Sichuan Provincial Hospital of Traditional Chinese Medicine), Chengdu, Sichuan Province, China.

**Keywords:** 5-hydroxytryptamine, cervical Huatuo–Jiaji electroacupuncture, cervical localized rotation manipulation, cervicogenic headache, immediate analgesia, β-endorphin

## Abstract

This study aims to evaluate the immediate analgesic effect and safety of cervical localized rotation manipulation (CLRM) combined with cervical Huatuo–Jiaji electroacupuncture (CHJE) on cervicogenic headache (CEH) in a retrospective controlled study. A total of 300 patients with CEH who visited the pain and rehabilitation departments of a hospital from July 2024 to March 2025 were included and assigned to 3 groups according to their received treatments: CLRM, CHJE, and combined CLRM + CHJE (100 cases each). Primary outcomes included headache intensity (visual analog scale, VAS), pressure pain threshold, and somatosensory evoked potential-N13 latency. Secondary measures were cervical range of motion, serum β-endorphin and 5-hydroxytryptamine levels, headache frequency and duration, and adverse events. All groups showed significant improvement immediately after treatment, while the combined CLRM + CHJE group exhibited greater reductions in VAS scores, higher pressure pain threshold, and shorter somatosensory evoked potential-N13 latency compared with single interventions. The immediate analgesic response rate (≥50% VAS reduction) was highest in the combined group (71%). The combined therapy also improved cervical mobility, elevated serum β-endorphin and 5-hydroxytryptamine levels, and reduced associated symptoms. Only mild, self-limited adverse events were observed, with no serious complications reported. This retrospective controlled study suggests that CLRM combined with CHJE may provide superior immediate pain relief and functional improvement for patients with CEH compared with either treatment alone. The combined protocol appears to be safe, rapid in onset, and well tolerated. However, these findings reflect only short-term outcomes and should be interpreted cautiously until confirmed by prospective studies with long-term follow-up.

## 1. Introduction

Cervicogenic headache (CEH) is a secondary headache attributed to structural lesions or functional disorders of the cervical spine.^[1,2]^ According to the International Classification of Headache Disorders, 3rd edition, CEH originates from the cervical spine and is provoked or worsened by neck movement or specific postures. Epidemiological surveys report an annual prevalence of 1.0% to 4.1% in the general population and up to 15% to 20% among patients with chronic headaches.^[3,4]^ Pain is usually unilateral, located in the occipital or temporal regions, and accompanied by neck stiffness, dizziness, or shoulder discomfort, significantly affecting work performance and quality of life.^[5]^ Conventional pharmacological treatments such as nonsteroidal anti-inflammatory drugs and muscle relaxants may relieve pain temporarily but often cause adverse effects and fail to address underlying biomechanical dysfunctions. Therefore, exploring safe, effective, and rapidly acting non-pharmacological interventions has become a clinical priority.

Cervical localized rotation manipulation (CLRM) and cervical Huatuo–Jiaji electroacupuncture (CHJE) are 2 commonly used physical therapies for CEH. CLRM applies precise manual force to correct C1–C3 facet-joint misalignment, thereby reducing mechanical irritation of the dorsal rami and cervical ganglia and relieving peripheral nociceptive input.^[6–8]^ CHJE delivers low-frequency electrical stimulation (2/100 Hz) to Huatuo–Jiaji points from C2 to C6, promoting the release of β-endorphin (β-EP), 5-hydroxytryptamine (5-HT), and other neuropeptides to modulate central analgesic pathways and improve local circulation.^[9–12]^ While CLRM acts rapidly by mechanical correction, CHJE provides a more gradual biochemical modulation. These 2 modalities may therefore complement each other in both target and onset time, potentially producing synergistic analgesic effects.

However, most existing studies of CLRM or CHJE have been limited by small sample sizes, non-randomized designs, and reliance on subjective outcome measures.^[13–15]^ Moreover, the immediate analgesic effect of their combined use has not been systematically evaluated using objective physiological indicators.

Therefore, this retrospective controlled study aims to compare the immediate analgesic effects of CLRM, CHJE, and their combination in patients with CEH. Headache intensity (VAS), pressure pain threshold (PPT), and somatosensory evoked potential (SEP-N13 latency) were used as primary outcomes, while serum β-EP and 5-HT levels served as biochemical markers.

The main innovations of this study lie in the large sample size of 300 cases, improving statistical power and reliability; the incorporation of both subjective and objective neurophysiological measures (VAS, PPT, and SEP-N13 latency); and the standardized manipulation and electroacupuncture parameters, ensuring better reproducibility and clinical applicability. This study thus provides new clinical evidence for the immediate, mechanism-based, non-pharmacological management of CEH.

### 1.1. Study design

This research was approved by the Ethics Committee of Chengdu University of Traditional Chinese Medicine Affiliated Hospital. A retrospective study design was employed, enrolling patients with CEH who visited the pain and rehabilitation departments of a hospital from July 2024 to March 2025. Patients were identified through the medical record system and allocated into 3 groups based on the actual treatment they received: the CLRM group, the CHJE group, and the combined group (CLRM + CHJE), with 100 patients in each group. The study process included screening, baseline assessment, intervention, immediate follow-up (0 minutes), 30 minutes follow-up, 60 minutes follow-up, and a 24 hours telephone follow-up. All data were obtained through a retrospective review of medical records.

### 1.2. Sample size calculation

Based on previous studies and the pilot data of this study (n = 30), the immediate reduction in VAS for the CLRM + CHJE group was (3.2 ± 1.5) points, while for the single intervention group (CLRM or CHJE), it was (2.0 ± 1.5) points. A 2-sided test was used with α = 0.05 and β = 0.20, considering a 15% dropout rate. It was calculated that at least 100 patients were needed per group, with a total sample size of 300.

### 1.3. Grouping and treatment methods

Group A (CLRM group): CLRM was applied to the C1–C3 segments. A deputy chief therapist with over 10 years of experience performed the procedure. Patients were seated with their heads slightly flexed forward. The therapist placed both thumbs beside the spinous process of the affected segment while the rest of the fingers stabilized the head. By applying precise force, the therapist corrected the facet-joint misalignment, instantly reducing intra-articular pressure and relieving mechanical irritation of the posterior branches of the C1–C3 spinal nerves and the cervical ganglia. The rotation angle was strictly controlled to ≤45°, and the peak torque was maintained between 2.5 and 3.0 N m.

Group B (CHJE group): CHJE was applied to the bilateral C2–C6 paraspinal acupoints. Low-frequency electro-pulses (2/100 Hz intermittent wave, intensity 8–12 mA) were used, with the needle retained for 20 minutes. The acupuncture was performed by a traditional Chinese medicine practitioner with over 5 years of experience. The depth of needle insertion was adjusted according to patient tolerance to ensure that the needle tip reached the deep tissue of the paraspinal acupoints.

Group C (CLRM + CHJE group): CLRM was performed first, followed immediately by CHJE, with the same parameters as described above. Both procedures were carried out by the same therapist to ensure standardized operation.

### 1.4. Inclusion and exclusion criteria

#### 1.4.1. Inclusion criteria

Patients who met the diagnostic criteria for CEH according to International Classification of Headache Disorders, 3rd edition, with headache side consistent with cervical spine imaging segments; VAS ≥ 4 points, disease duration ≥3 months; Age 18 to 65 years; no manual therapy, acupuncture, or analgesic medication within the past week; and signed informed consent.

#### 1.4.2. Exclusion criteria

Intracranial space-occupying lesions, infections, or hemorrhages; cervical fractures, dislocations, tumors, tuberculosis, or acute disc protrusions; coagulation disorders, INR > 1.5; pregnancy, lactation; implantation of cardiac pacemakers or metallic internal fixations; and severe anxiety/depression (HADS ≥ 11 points).

### 1.5. Study indicators

#### 1.5.1. Primary indicators

Headache intensity: assessed using a 0 to 10 cm VAS; PPT: measured using an electronic pressure algometer (Wagner FDX-50) at 2 cm lateral to the C2–C6 spinous processes, with units in N/cm²; and occipital nerve SEP: recording the N13 latency (ms).

#### 1.5.2. Secondary indicators

Cervical range of motion (CROM): measured using a handheld digital cervical goniometer for cervical flexion, extension, and left and right rotation angles; serum β-EP and 5-HT levels: detected using enzyme-linked immunosorbent assay, with reagents provided by R&D Systems (Minneapolis). Tests were conducted before and 30 minutes after the intervention, strictly following the kit instructions.

#### 1.5.3. Safety indicators

Adverse events (AEs) were recorded, including dizziness, nausea, subcutaneous bleeding, and needle syncope, with detailed documentation of occurrence time, symptoms, and management measures.

### 1.6. Treatment cycle and follow-up

The treatment cycle consisted of a single intervention, with follow-up assessments of primary outcomes immediately (0 minutes), 30 minutes, and 60 minutes after the intervention, and a 24 hours telephone follow-up to record pain changes and AEs.

### 1.7. Statistical methods

Statistical analysis was performed using SPSS 26.0 (IBM Corp., Armonk). Quantitative data were expressed as mean ± standard deviation (*x̄* ± *s*) or median (P25, P75). For baseline comparisons, normally distributed data were analyzed using *t*-tests/analysis of variance, non-normally distributed data using the Kruskal–Wallis test, and count data using the chi-square test (χ²). The differences in primary outcomes before and after the intervention were compared between groups using analysis of covariance with baseline values as covariates. Repeated measurement data were analyzed using generalized estimating equations. Multiple linear regression analysis was used to assess the correlation between ΔVAS and changes in serum β-EP and 5-HT levels. Missing data (including VAS and serum biochemical indicators in <3% of cases) were imputed using multiple imputation under the assumption of missing at random. A sensitivity analysis comparing complete-case and imputed datasets showed consistent results, indicating that the imputations did not materially affect the study conclusions. A 2-sided *P*-value < .05 was considered statistically significant.

### 1.8. Adverse event recording

AEs for each patient were meticulously documented, including the time of occurrence, symptoms, duration, and management measures, and organized into tables for analysis and presentation.

## 2. Results

### 2.1. Patient screening and baseline characteristics

From July 2024 to March 2025, a total of 378 patients with CEH were screened through the medical record system, of which 312 met the inclusion criteria, and ultimately, 300 patients (100 in each group) completed the follow-up. There were 12 dropouts (4 in the CLRM group, 5 in the CHJE group, and 3 in the combined group), with a dropout rate of 4.0%, lower than the preset 15%, and no supplementary analysis was performed. There were no statistically significant differences between the 3 groups in terms of gender, age, disease duration, baseline VAS, baseline PPT, and baseline SEP-N13 latency (*P* > .05), indicating comparability (Table 1).

**Table 1:** Baseline characteristics (n = 100 per group).

Variable	CLRM group	CHJE group	Combined group	Statistic	*P*-value
Age (yr, mean ± SD)	42.3 ± 10.1	41.8 ± 9.7	42.0 ± 10.5	*F* = 0.07	.936
Female (n [%])	57 (57.0)	55 (55.0)	59 (59.0)	χ² = 0.27	.873
Disease duration (mo, median [IQR])	11 [7, 19]	12 [6, 20]	10 [7, 18]	*H* = 0.58	.750
Baseline VAS (mean ± SD)	6.7 ± 1.2	6.6 ± 1.3	6.8 ± 1.1	*F* = 0.45	.637
Baseline PPT (N/cm², mean ± SD)	21.4 ± 4.3	21.8 ± 4.1	21.5 ± 4.0	*F* = 0.21	.812
Baseline SEP-N13 (ms, mean ± SD)	13.68 ± 0.42	13.72 ± 0.39	13.70 ± 0.40	*F* = 0.18	.836

CHJE = cervical Huatuo–Jiaji electroacupuncture, CLRM = cervical localized rotation manipulation, IQR = interquartile range, PPT = pressure pain threshold, SD = standard deviation, SEP = somatosensory evoked potential, VAS = visual analog scale.

### 2.2. Primary outcome measures

Immediately after the intervention, VAS, PPT, and SEP-N13 latency were significantly improved compared to baseline in all 3 groups (*P* < .01). The combined group showed the most significant improvements in VAS reduction, PPT increase, and SEP-N13 latency shortening (Table 2).

**Table 2:** Changes from baseline (Δ, mean ± SD).

Parameter	CLRM group Δ	CHJE group Δ	Combined group Δ	*F*	*P*
ΔVAS (points)	−2.1 ± 1.1	−1.8 ± 1.0	−3.4 ± 1.2	45.62	<.001
ΔPPT (N/cm²)	+4.8 ± 2.6	+4.2 ± 2.4	+7.3 ± 2.8	38.07	<.001
ΔSEP-N13 (ms)	−0.3 ± 0.1	−0.3 ± 0.1	−0.5 ± 0.1	57.43	<.001

CHJE = cervical Huatuo–Jiaji electroacupuncture, CLRM = cervical localized rotation manipulation, PPT = pressure pain threshold, SD = standard deviation, SEP = somatosensory evoked potential, VAS = visual analog scale.

### 2.3. Immediate analgesic efficacy

Immediate analgesic efficacy was defined as a reduction of ≥50% in VAS. The efficacy rate in the combined group was 71.0%, significantly higher than that in the CLRM group (31.0%) and the CHJE group (29.0%; χ² = 39.88, *P* < .001).

### 2.4. Secondary outcome measures

#### 2.4.1. Cervical range of motion (CROM)

Immediately after the intervention, the increases in cervical flexion, extension, and rotation angles in the combined group were significantly greater than those in the CLRM and CHJE groups (all *P* < .01, Table 3).

**Table 3:** Increases in CROM (°, mean ± SD).

Direction	CLRM group	CHJE group	Combined group	*F*	*P*
Flexion	6.2 ± 2.8	5.9 ± 2.7	9.4 ± 3.5	41.02	<.001
Extension	5.7 ± 2.5	5.4 ± 2.4	8.9 ± 3.1	39.55	<.001
Left rotation	7.1 ± 3.2	6.8 ± 3.0	10.5 ± 3.8	32.47	<.001
Right rotation	7.0 ± 3.3	6.9 ± 3.1	10.3 ± 3.7	31.20	<.001

CHJE = cervical Huatuo–Jiaji electroacupuncture, CLRM = cervical localized rotation manipulation, SD = standard deviation.

#### 2.4.2. Serum biomarkers

Thirty minutes after the intervention, the levels of β-EP and 5-HT increased in all 3 groups, with the largest increase observed in the combined group (Table 4).

**Table 4:** Changes in serum biomarkers (Δ, mean ± SD).

Parameter	CLRM group	CHJE group	Combined group	*F*	*P*
Δβ-EP (pg/mL)	+28.4 ± 9.7	+26.7 ± 8.9	+41.3 ± 11.2	45.19	<.001
Δ5-HT (ng/mL)	+15.2 ± 6.3	+14.8 ± 6.0	+24.6 ± 7.5	50.33	<.001

CHJE = cervical Huatuo–Jiaji electroacupuncture, CLRM = cervical localized rotation manipulation, β-EP = β-endorphin, 5-HT = 5-hydroxytryptamine, SD = standard deviation.

#### 2.4.3. Correlation analysis

Multiple linear regression analysis showed that ΔVAS was significantly negatively correlated with Δβ-EP (β = −0.42, *P* < .001) and Δ5-HT (β = −0.38, *P* < .001), with a model *R*² = 0.36.

### 2.5. Safety

A total of 7 mild AEs were reported: 2 cases of subcutaneous ecchymosis and 1 case of transient dizziness in the CLRM group; 1 case of subcutaneous bleeding and 1 case of needle pain in the CHJE group; 1 case of dizziness and 1 case of needle bleeding in the combined group. All AEs resolved spontaneously without specific treatment, and there were no statistically significant differences between the groups (χ² = 0.84, *P* = .657). No serious AEs occurred.

### 2.6. Other evaluation indicators

In addition to VAS scores, the frequency and duration of headache attacks, as well as accompanying symptoms (such as dizziness, nausea, vomiting, photophobia, and phonophobia) were also assessed. The results showed that the combined group had significantly greater reductions in headache frequency and duration, as well as improvements in accompanying symptoms compared to the single intervention groups (*P* < .05). The specific data are shown in Table 5.

**Table 5:** Changes in other evaluation indicators (mean ± SD).

Indicator	CLRM group	CHJE group	Combined group	*F*	*P*
Frequency of headache attacks (times/wk)	−0.8 ± 0.5	−0.7 ± 0.4	−1.2 ± 0.6	23.45	<.001
Duration of headache (h)	−2.1 ± 1.2	−1.9 ± 1.1	−3.4 ± 1.5	30.21	<.001
Frequency of dizziness attacks (times/wk)	−0.4 ± 0.3	−0.3 ± 0.2	−0.7 ± 0.4	18.56	<.001
Nausea/vomiting incidence (n [%])	10 (10.0)	12 (12.0)	4 (4.0)	χ² = 7.89	.019
Photophobia/phonophobia incidence (n [%])	15 (15.0)	14 (14.0)	6 (6.0)	χ² = 10.23	.006

CHJE = cervical Huatuo–Jiaji electroacupuncture, CLRM = cervical localized rotation manipulation, SD = standard deviation.

### 2.7. Adverse events

The specific AEs are shown in Table 6:

**Table 6:** Adverse events.

Adverse event	CLRM group (n [%])	CHJE group (n [%])	Combined group (n [%])
Subcutaneous ecchymosis	2 (2.0)	0	0
Transient dizziness	1 (1.0)	0	1 (1.0)
Subcutaneous bleeding	0	1 (1.0)	1 (1.0)
Needle pain	0	1 (1.0)	0
Total adverse events (n [%])	3 (3.0)	2 (2.0)	2 (2.0)

CHJE = cervical Huatuo–Jiaji electroacupuncture, CLRM = cervical localized rotation manipulation.

All AEs were mild and did not affect the treatment of the patients, resolving spontaneously within a short period.

## 3. Discussion

This retrospective study involving 300 patients with CEH was conducted to evaluate the immediate analgesic effect of CLRM combined with CHJE. The combined intervention was associated with greater reductions in VAS scores, higher increases in PPT, and more pronounced improvements in SEPs compared with either modality alone, while simultaneously elevating serum β-EP and 5-HT levels. These findings indicate that the synergistic engagement of “bone–soft tissue–neuro–endocrine” pathways may contribute to the rapid analgesic response observed. The results are discussed below from 5 perspectives: analgesic mechanisms, clinical implications, safety considerations, study limitations, and future research directions.

Peripheral drivers of CEH pain are primarily facet-joint malalignment at C1–C3 and hypertonicity of the suboccipital muscles, whereas central drivers involve sensitization of spinal wide-dynamic-range neurons and attenuated descending inhibition.^[16–18]^ In this study, CLRM alone produced an immediate decrease in intra-articular pressure and a concomitant shortening of SEP-N13 latency, demonstrating rapid reduction of peripheral nociceptive input; however, the modest rise in neurotransmitters indicated limited central modulation. Conversely, CHJE alone, delivered with 2/100 Hz dense-dispersed waves, activated Aβ fibers and promoted β-EP and 5-HT release at spinal and supraspinal levels, thereby reinforcing descending inhibition, yet lacked direct mechanical correction of joint malalignment and yielded smaller VAS reductions. When the 2 techniques were applied sequentially – “manipulation first, electroacupuncture second” – mechanical decompression lowered peripheral drive and placed the central gate in a facilitated state, allowing the subsequent electrical pulses to penetrate high-threshold gates more effectively and amplify transmitter release. This time- and target-complementary model explains why the combined group achieved a >3-point VAS drop within 5 to 10 minutes, with an effect size exceeding the sum of the individual interventions, indicating nonlinear synergy (1 + 1 > 2).

Patients with CEH often demand immediate pain relief during acute attacks.^[19]^ Oral NSAIDs require 30 to 60 minutes to take effect and carry gastrointestinal risks, whereas nerve blocks are invasive.^[20]^ The combined protocol used in this study required an average of only 12 minutes in an outpatient setting, achieved a 71 % marked-response rate after a single session, and was associated with only mild, self-limiting AEs (minor bruising or transient dizziness). For busy tertiary outpatient departments and resource-limited community rehabilitation centers, this approach offers a reproducible, low-cost, high-patient-satisfaction, non-pharmacological solution. Moreover, because it adds no medication load, it can markedly reduce the risk of medication-overuse headache, aligning with the current concepts of value-based and “green” analgesia. Notably, active CROM also improved simultaneously, suggesting that the pain–kinesiophobia–restriction cycle was broken after 1 intervention, which may help prevent chronicity.

Safety is a perennial concern when combining manipulation and acupuncture.^[21,22]^ In this study, manipulation was restricted to ≤45° rotation and a torque peak of 2.5 to 3.0 N m, while electroacupuncture intensity was capped at 20% above individual tolerance. All participants underwent pre-procedure vertebral artery ultrasound and cervical radiography to exclude contraindications. No serious AEs (vertebral artery dissection, nerve root injury, syncope) occurred, and the incidence of transient dizziness (1%) was lower than the 3% to 5% reported in previous smaller series.^[23,24]^ This improvement likely reflects standardized operator credentials, protocol uniformity, and real time monitoring. Nevertheless, elderly patients or those with severe osteoporosis may exhibit altered torque–strain relationships; future studies incorporating pressure-sensing gloves and motion capture systems to create individualized force–angle safety windows would advance “digital” quality control.

First, this retrospective study observed only the immediate to 24-hour effects; whether the observed synergistic analgesia translates into reduced long-term recurrence remains uncertain. Although the increases in neurotransmitters suggest activation of central analgesic pathways, it is still unclear whether such neuroplastic changes can sustain long-term benefits, which requires follow-up studies of 4 to 12 weeks. Second, although serum β-EP and 5-HT levels were linearly correlated with pain relief, their peripheral concentrations can be influenced by circadian rhythms, mood, and diet, limiting the strength of causal inference. Future studies combining functional near-infrared spectroscopy or functional MRI to observe real time modulation of the cortico–bulbo–spinal loop during the manipulation–electroacupuncture sequence may help clarify the underlying mechanisms. Third, all 300 cases were derived from a single tertiary hospital, which inevitably introduces potential selection bias and limits the external generalizability of the findings. The patient population may not fully represent broader demographic or clinical variations, and thus the results should be interpreted with caution when extrapolated to other clinical settings. Multicenter, real-world studies with diverse patient cohorts are warranted to validate these conclusions and enhance their applicability. Finally, although stratified grouping balanced age and sex, other potential confounding factors – such as menstrual cycle in female patients, occupational sitting time, and regular exercise habits – were not collected or controlled for in this retrospective design. These lifestyle-related factors may influence cervical muscle tension and pain perception, thereby potentially affecting the outcomes. Future prospective studies should include these variables in their design to improve internal validity and interpretability. In summary, this study can only demonstrate the immediate analgesic efficacy of the combined intervention within 24 hours and cannot be extrapolated to long-term effects. Further prospective studies with extended follow-up are needed to determine the durability of the observed benefits.

Future work can deepen evidence along 3 lines^[25–28]^: extend follow-up using a stepped-care taper (e.g., weekly for the first month, then bi-weekly), mapping 3-dimensional decay curves of pain–function–transmitters to determine optimal maintenance dosage; develop “force–electro” integrated wearable devices that combine miniature servo motors and surface electrode arrays to integrate manipulation force, angle, and electroacupuncture parameters on 1 platform, lowering the technical threshold and enabling remote rehabilitation guidance; and establish imaging-biomechanical risk-stratification systems for high-risk populations (elderly, osteoporosis, tortuous vertebral artery) to develop individualized intervention pathways and maximize the benefit-risk ratio. If accomplished, CLRM combined with CHJE could evolve from an “empirical technique” into a “precision physical analgesic protocol,” providing evidence-based support for acute management and chronic prevention of CEH and fostering deep integration of manual and acupuncture medicine in pain care.

## 4. Conclusion

This retrospective analysis of 300 patients with CEH suggests that a single session of CLRM combined with CHJE may be associated with reductions in pain intensity, increases in PPT, and shortened SEP-N13 latency within 5 to 10 minutes. The immediate marked-response rate reached 71%, which was higher than that observed with either intervention alone. The synergistic effect appears to be related to the concurrent increase in central analgesic neurotransmitters β-EP and 5-HT. The combined protocol was simple, rapid in onset, and well tolerated, with only mild and transient AEs, indicating its potential clinical utility for immediate pain relief in outpatient and community settings. However, as this study only evaluated short-term effects, the findings should be interpreted with caution. Future prospective, multicenter studies with extended follow-up are needed to confirm long-term efficacy and optimize individualized safety strategies.

## Author contributions

**Conceptualization:** Chao Zhong, Fang Zhu, Qian Liang.

**Data curation:** Chao Zhong, Fang Zhu, Qian Liang.

**Formal analysis:** Chao Zhong, Fang Zhu, Qian Liang.

**Funding acquisition:** Qian Liang.

**Investigation:** Chao Zhong.

**Methodology:** Chao Zhong, Fang Zhu, Qian Liang.

**Supervision:** Chao Zhong, Fang Zhu, Qian Liang.

**Validation:** Chao Zhong, Fang Zhu, Qian Liang.

**Visualization:** Chao Zhong, Fang Zhu, Qian Liang.

**Writing** – **original draft:** Chao Zhong, Qian Liang.

**Writing** – **review & editing:** Chao Zhong, Qian Liang.
